# Disguising the Taste of Epsom Salts

**Published:** 1880-08

**Authors:** 


Disguising the Taste of Epsom Salt. — According to the "Gaz. des Hop," June 12, 1880, the *purgatif Yvon* consists of sulphate of magnesia 20 grams, water 40 grams, and essence of mint two or three drops. The essence of mint completely masks the disagreeable taste of the sulphate, providing that the quantity of the vehicle is inconsiderable.—*Medical and Surgical Reporter.*

### METEOROLOGICAL REPORT FOR AUGUST, 1880.

| RAIN. DATE | RAIN. IN. | Mean daily Barometer. | Mean daily Thermometer | Mean daily Humidity. | Maximum Thermometer | Minimum Thermometer | Prevailing direction of Wind. | Maximum hourly velocity of Wind, (Daily.) |
|---|---|---|---|---|---|---|---|---|
| 1 | 0 | 30.083 | 77.7 | 58.0 | 86 | 69 | S. E. | ......... |
| 2 | 0 | 29 927 | 80.7 | 56.7 | 87 | 71 | S E. | ......... |
| 3 | 78 | 29.866 | 76.0 | 76 0 | 85 | 71 | S. | ......... |
| 4 | 70 | 29.909 | 74.5 | 82.7 | 81 | 71 | N. W. | ......... |
| 5 | 95 | 29.998 | 72.2 | 86.7 | 80 | 68 | N. E. | ......... |
| 6 | 47 | 30 129 | 65.7 | 91.3 | 69 | 64 | N. E. | ......... |
| 7 | 0 | 30.120 | 68.5 | 81.0 | 73 | 65 | N. E. | ......... |
| 8 | — | 29.986 | 71.0 | 85.0 | 77 | 66 | E. | ......... |
| 9 | 0 | 29.992 | 78.2 | 72.7 | 85 | 68 | S. E. | ......... |
| 10 | 08 | 30 067 | 73.5 | 82.7 | 84 | 69 | S. | ......... |
| 11 | 01 | 30.040 | 75.2 | 80.3 | 82 | 70 | S. W. | ......... |
| 12 | 0 | 30.027 | 77.0 | 73.0 | 86 | 69 | N. W. | ......... |
| 13 | 0 | 29.970 | 76.0 | 77.0 | 84 | 68 | N. E. | ......... |
| 14 | 0 | 29.991 | 78.2 | 60.7 | 85 | 67 | E. | ......... |
| 15 | 0 | 30.141 | 81.0 | 53.0 | 87 | 71 | N. W. | ......... |
| 16 | 0 | 30.218 | 80.5 | 59.3 | 87 | 72 | N. E. | ......... |
| 17 | 0 | 30.136 | 76.7 | 73 7 | 83 | 69 | E. | ......... |
| 18 | 0 | 29.999 | 72.5 | 83 0 | 81 | 68 | E | ......... |
| 19 | 0 | 29.863 | 75.5 | 79.3 | 82 | 68 | N. | ......... |
| 20 | 0 | 29.815 | 81.5 | 64.7 | 88 | 72 | W. | ......... |
| 21 | 0 | 29.887 | 83.2 | 63 0 | 88 | 74 | W. | ......... |
| 22 | 03 | 29.965 | 81.5 | 58.3 | 91 | 74 | N. W. | ......... |
| 23 | 0 | 30 014 | 82 5 | 64.3 | 92 | 71 | N. E. | ......... |
| 24 | 03 | 29.993 | 78.2 | 74 0 | 91 | 71 | N. E. | ......... |
| 25 | 08 | 29.979 | 75.5 | 80.3 | 86 | 70 | S. W | ......... |
| 26 | 04 | 30.013 | 78.0 | 72.3 | 87 | 69 | S. E. | ......... |
| 27 | 12 | 30 060 | 76.2 | 82.0 | 84 | 69 | E. | ......... |
| 28 | 0 | 29.990 | 76.2 | 77.3 | 82 | 71 | E. | ......... |
| 29 | 0 | 29.894 | 78.2 | 71 0 | 85 | 69 | N. E. | ......... |
| 30 | — | 29.811 | 76.0 | 76.0 | 85 | 70 | N. E. | ......... |
| 31 | 32 | ......... .. | 75.0 | 86.7 | 83 | 71 | E. | ......... |
| Sums * | | 29.998 | 76 5 | 73.3 | 84.0 | 69.5 | ......... | ......... |

*Not enough to measure.

Highest Barometer, 30.278, on the 7th.
Lowest Barometer, 29.741, on the 31st.
Monthly range of Barometer, 0.537.
Highest Temp. 92°, on 23d. Lowest Temp. 64°, on 6th.
Monthly range of Temperature, 28°.
Greatest daily range of Temperature, 21°, on the 23d.
Least daily range of Temperature, 5°, on the 6th.
Mean of Maximum Temperatures, 84°.0.
Mean of Minimum Temperatures, 69°.5.
Total rainfall or melted snow, 3.61 inches.
Prevailing wind, East. Total movement of wind, 53.03 miles.
Maximum velocity of wind and direction, 38 miles per hour; east, on 31st.
Number of foggy days, 0
Number of clear days, 2.
Number of fair days, 9.
Number of cloudy days on which rain or snow fell, 11.
Number of cloudy days on which no rain or snow fell, 7.
Total number of days on which rain fell, 14.

H. HALL, Corp'l. Signal Service U. S. A

Atlanta, Ga., August, 1880.

